# The *Aurantii Fructus Immaturus* flavonoid extract alleviates inflammation and modulate gut microbiota in DSS-induced colitis mice

**DOI:** 10.3389/fnut.2022.1013899

**Published:** 2022-10-05

**Authors:** Si-Yuan Chen, Qing Yi-Jun Zhou, Lin Chen, Xin Liao, Ran Li, Tao Xie

**Affiliations:** ^1^The First Hospital of Hunan University of Chinese Medicine, Hunan University of Chinese Medicine, Changsha, China; ^2^Science and Technology Innovation Center, Hunan University of Chinese Medicine, Changsha, China; ^3^Institute of Chinese Materia Medica, Hunan Academy of Chinese Medicine, Changsha, China; ^4^Guangdong Provincial Key Laboratory of Utilization and Conservation of Food and Medicinal Resources in Northern Region, Shaoguan University, Shaoguan, China; ^5^Hunan Yueyang Maternal & Child Health-Care Hospital, Yueyang, China; ^6^Changsha Traditional Chinese Medicine Hospital, Changsha, China

**Keywords:** *Aurantii Fructus Immaturus*, intestinal flora, anti-inflammatory, neohesperidin, naringin

## Abstract

Inflammatory bowel disease (IBD) is a chronic, relapsing immune-mediated disease that always leads to a progressive loss of intestinal function. Therefore, it is important to find potential therapeutic drugs. This study was conducted to elucidate the effect of *Aurantii Fructus immaturus* flavonoid extract (AFI, 8% neohesperidin, 10% naringin) on DSS-induced intestinal inflammation and the gut microbiome. To explore the mechanism of action by which AFI protects against intestinal inflammation, a total of 50 mice were randomly divided into 5 groups [CG (control group), MG (model group), AFI low dose, AFI middle dose, and AFI high dose] and received 2.5% DSS for 7 days. Then, mice in the AFI groups were orally administered different doses of AFI for 16 days. The results showed that, compared with the MG group, the food intake and body weight were increased in the AFI groups, but the water intake was lower. Additionally, AFI significantly alleviated DSS-induced colitis symptoms, including disease activity index (DAI), and colon pathological damage. The levels of IL-6, IL-1β and TNF-α in serum and colon tissue were significantly decreased. The diversity and abundance of the intestinal microbiota in the AFI group were decreased. The relative abundance of Bacteroidota was increased, and the relative abundance of Firmicutes was decreased. AFI plays an important role in alleviating DSS-induced intestinal inflammation and regulating Oscillospira, Prevotellaceae and Lachnospiraceae in the intestine at low, medium and high doses, respectively. This report is a pioneer in the assessment of AFI. This study not only demonstrated the anti-inflammatory activity of AFI but also identified the microbiota regulated by different concentrations of AFI.

## Introduction

Inflammatory bowel disease (IBD) is a type of chronic, relapsing immune-mediated disease that always leads to the progressive loss of intestinal function (1). IBD has long been a huge challenge and burden on the public health care system (2, 3). In fact, IBD is caused by multiple pathogens and multiple pathogens, and there is no effective treatment (4). Therefore, finding a useful drug has become an urgent problem that needs to be solved.

*Aurantii Fructus Immaturus* (Chinese name Zhishi) is the dried unripe fruit of *Citrus aurantium* L. or its cultivars or *Citrus sinensis Osbeck* that is collected from May to June. For a long time, the processed unripe fruits of Bittet Orange, possesses homology of medicine and food characteristic, which is regarded to be health promotion effect in digestive tract system. Additionally, *Aurantii Fructus Immaturus* has been used as a single Chinese medicine and compound to treat gastrointestinal diseases, such as diarrhea, gasteremphraxis and uterine prolapse. The active ingredients of *Citrus aurantium*, such as flavonoids, alkaloids, volatile oils and coumarins, have antifungal (5), anti-anxiety (6), antioxidant (7), anticancer (8), anti-inflammatory (9), gastric mucosa protective (10), anticoagulation, intestinal motility regulatory (11), nerve protective (12) and other effects. In previous studies, some single compound extracted from *Aurantii Fructus Immaturus*, such as neohesperidin and naringin have reported can relieve inflammation (13).

In the present study, we investigated the effects of *Aurantii Fructus immaturus* flavonoid extract (AFI, neohesperidin 8%, naringin 10%) on dextran sulfate sodium (DSS)-induced intestinal inflammation and the gut microbiome. We demonstrate that AFI treatment alleviates DSS-induced gut inflammation and suggest that it is related to the regulation of the intestinal microbiota. These findings thus demonstrate that AFI represents a potential agent for the treatment of intestinal inflammation.

## Materials and methods

### Chemicals and reagents

The *Aurantii Fructus Immaturus* flavonoid extract (neohesperidin 8% and naringin 10%) was purchased from Kanglu Biotechnology Co., Ltd. (Hunan Province, China). Mouse interleukin 6 (IL-6) (#MM-0163M1), tumor necrosis factor (TNF-α) (#MM-0132M1) and interleukin 1β (IL-1β) (#MM-0040M1) enzyme-linked immunosorbent assay (ELISA) kits were obtained from Jiangsu Meimian Industrial Co. Ltd. (Jiangsu, China). The RNA extraction kit was obtained from Aidlab Biotechnologies Co. Ltd. (Beijing, China). The iScript cDNA synthesis kit and SYBR Green master mix were obtained from Bio-Rad Laboratories, Inc. (Hercules, CA, USA).

### Animals and drug administration

The animal research was conducted according to the Guidelines for Animal Experimentation of Hunan University of Chinese Medicine (Changsha, China) and was approved by the Animal Ethics Committee of Hunan University of Chinese Medicine. A total of 50 C57BL/6J male mice (8 weeks old and 18 to 20 g) were obtained from Hunan SJA Laboratory Animal Co., Ltd. (Hunan, China). The mice were acclimated for 1 week. During this time, the mice were fed standard food and given free access to water. Then, they were randomly divided into 5 groups [CG (control group), MG (model group), AFIL (AFI low dose at 50 mg/kg), AFIM (AFI middle dose at 100 mg/kg), and AFIH (AFI high dose at 200 mg/kg); *n* = 10/group], and the mice received 2.5% DSS in their drinking water for 7 days. All doses of AFIs (AFIL, AFIM and AFIH) were administered by gavage once a day, starting from the 8th day of the experiment. The MG and CG mice were gavaged with saline. Moreover, we used the DAI data which presented as an average score of the diarrhea of stool, body weight loss, and the extent of blood in the feces to evaluated the severity of colitis by this scoring system (14). On the 23rd day, the mice were sacrificed with CO2, and blood was collected via the cardiac puncture method. All mice were observed once a day, and the food intake and water intake by body weight were recorded. The colon samples embedded in paraffin were cut into 4 μm slices. Sections were stained with hematoxylin and eosin (H&E). Histological damage of the colons was observed in H&E-stained sections using a light mi- croscope, and histopathological scores were evaluated according to the scoring system (15). Then, the colon tissue and serum were collected for subsequent qRT-PCR experiments. The intestinal contents were collected, snap-frozen in liquid nitrogen and then stored at –80°C for further analysis.

### Real-time quantitative polymerase chain reaction

Total RNA of the colon tissues was extracted using TRIzol reagent (Life Technologies, USA) according to the manufacturer’s instructions. Then, the total RNA concentrations were equalized (1 μg) and converted to cDNA using the iScript cDNA synthesis kit according to the manufacturer’s protocol. Gene expression was measured by quantitative polymerase chain reaction (qPCR) using SYBR Green Master Mix (Roche, Basel, Switzerland) on a CFX96 Real-Time PCR system from Bio-Rad. Gene expression was measured by qPCR (Roche, Basel, Switzerland) using SYBR Green (Roche, Basel, Switzerland). GAPDH or β-actin was used for gene expression normalization in animal tissue or cells, respectively. The relative expression levels of genes were calculated using the 2^–ΔΔCt^ method. The primers are listed in the Supplementary material (Supplementary Table 1).

### Gut microbiota profiling 16S rDNA amplicon sequencing

Genomic DNA was extracted from the intestinal content samples using the E.Z.N. Stool DNA kit. The hypervariable region V3-V4 of the bacterial 16S rRNA gene was amplified with primer pair 338F (5’-ACTCCTACGGGAGGCAGC AG-3’). The genomic DNA from the fecal samples was extracted using a DNA kit (TIANGEN Biotech Co. Ltd., Beijing, China) and quantified using a Qubit 2.0 fluorometer (Invitrogen, Carlsbad, CA, USA). DNA (30 to 50 ng) was used to generate amplicons using a MetaVx Library Preparation kit. The V3 and V4 hypervariable regions of prokaryotic 16S rDNA were selected for generating amplicons and subsequent taxonomy analysis. DNA libraries were validated by an Agilent 2100 Bioanalyzer (Agilent Technologies, Palo Alto, CA, USA) and quantified by Qubit 2.0 Fluorometer. DNA libraries were multiplexed and loaded on an Illumina MiSeq instrument according to the manufacturer’s instructions (Illumina, San Diego, CA, USA). Sequencing was performed by Majorbio Bio-Pharm Technology using paired-end configuration; image analysis and base-calling were conducted by the MiSeq Control Software (MCS) embedded in the MiSeq instrument.

The raw 16S rRNA gene sequencing reads were demultiplexed, quality-filtered by fastp version 0.20.0 (16) and merged by FLASH version 1.2.7 (17) with the following criteria: (i) the 300 bp reads were truncated at any site receiving an average quality score of <20 over a 50 bp sliding window, and the truncated reads shorter than 50 bp were discarded, reads containing ambiguous characters were also discarded; (ii) only overlapping sequences longer than 10 bp were assembled according to their overlapped sequence. The maximum mismatch ratio of overlap region is 0.2. Reads that could not be assembled were discarded; (iii) Samples were distinguished according to the barcode and primers, and the sequence direction was adjusted, exact barcode matching, 2 nucleotide mismatch in primer matching. Operational taxonomic units (OTUs) with 97% similarity cutoff (18, 19) were clustered using UPARSE version 7.1 (19), and chimeric sequences were identified and removed. The taxonomy of each OTU representative sequence was analyzed by RDP Classifier version 2.2 (20) against the 16S rRNA database using confidence threshold of 0.7.

### Statistical analysis

The data are presented as the means ± SDs, and statistical analysis was performed using GraphPad Prism (USA). Datasets that involved more than two groups were assessed by one-way or two-way analysis of variance (ANOVA) followed by Tukey’s multiple comparison test. The Wilcoxon rank sum test and Tukey’s test were used to analyze the differences in species diversity between groups.

## Results

### *Aurantii Fructus immaturus* flavonoid extract ameliorates colitis induced by dextran sulfate sodium

**FIGURE 1:**
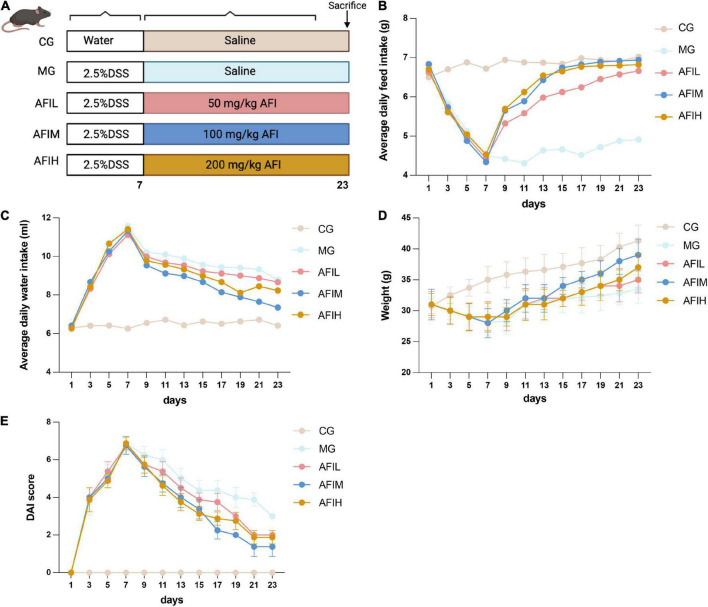
The effects of AFI on mice. **(A)** The experimental design, **(B)** food intake, **(C)** water intake, **(D)** body weights and **(E)** DAI scores for all mice.

The body weight of mice was remarkably reduced after DSS induction. However, this loss was reversed by AFI, and the colitis induced by DSS treatment was ameliorated (Figure 1A). We monitored the body weight, food intake and water intake of mice in the CG, MG, AFIL, AFIM, and AFIH groups. In the MG, AFIL, AFIM, and AFIH groups, the food intake and body weight gain were significantly decreased after 7 days of treatment with 2.5% DSS compared to the CG group; however, the water intake of mice was increased. After 16 days of AFI treatment, the body weight, food intake and water intake returned to levels close to those of the CG group and compared to those of the MG group (Figures 1B–D). In addition, the DAI scores of AFI groups were significantly decreased compared to the MG group (Figure 1E).

### The effect of *Aurantii Fructus immaturus* flavonoid extract on serum inflammatory factors

**FIGURE 2:**
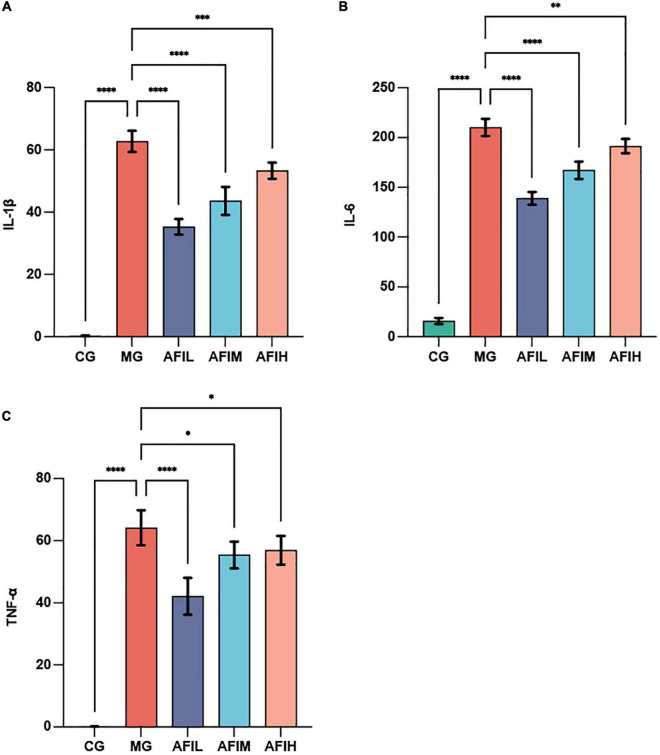
The effect of AFI on serum inflammatory factors. Bars represent the mean ± SE (*N* = 6). **(A)** Serum IL-1β levels. **(B)** Serum IL-6 levels. **(C)** The TNF-α level in the serum. The value of *P* < 0.05 is marked with “*”. The value of *P* < 0.01 is marked with “**”. The value of *P* < 0.005 is marked with “***”. The value of *P* < 0.001 is marked with “****”.

We found that the serum concentrations of IL1-β, IL-6 and TNF-α in the MG group were significantly higher than those in the CG and AFIL groups (*p* < 0.001; Figures 2A–C). Additionally, the serum IL1-β (*p* < 0.001; Figure 2A), IL-6 (*p* < 0.001; Figure 2A) and TNF-α (*p* < 0.05; Figure 2A) contents in the AFIM group were also significantly lower than those in the MG group. The levels of IL1-β (*p* < 0.005, Figure 2A), IL-6 (*p* < 0.01, Figure 2B) and TNF-α (*p* < 0.05, Figure 2C) in the serum of the AFIH group were significantly lower than those in the MG groups.

### The effect of *Aurantii Fructus immaturus* flavonoid extract on intestinal inflammatory factors

**FIGURE 3:**
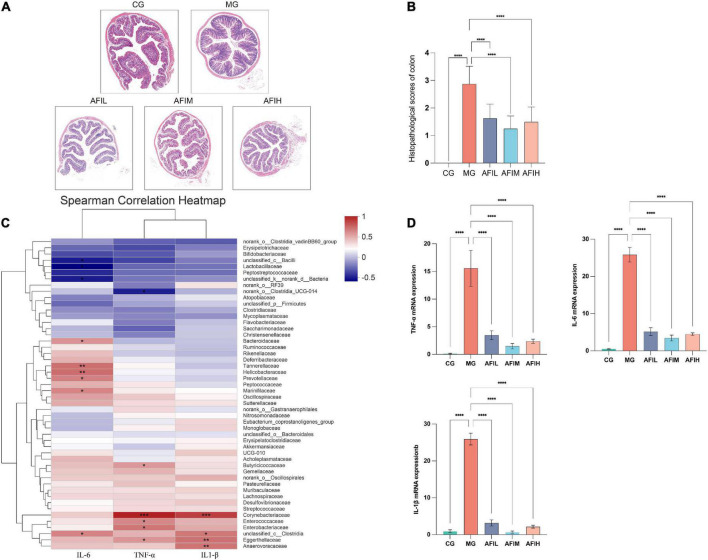
Histopathological, Intestinal inflammatory factors and Spearman correlation heatmap in all mice. **(A)** The colons of each group were processed for histological evaluation (H&E staining, 50×). **(B)** Histopathological scores of each group were evaluated. Data are presented as means ± SD (*n* = 8). ***p < 0.001, compared with the control group; * *p* < 0.05 and ** *p* < 0.01, compared with the MG group. **(C)** Correlation heatmap of IL-6, TNF-a, IL-1 and the microbiota. The X and Y axes are the environmental factors and families, respectively. R is shown in different colors. The right side of the legend is the color range of the different R values. The value of *P* < 0.05 is marked with “*”. The value of *P* < 0.01 is marked with “**”. The value of *P* < 0.005 is marked with “***”. The value of *P* < 0.001 is marked with ****. **(D)** IL-6, TNF-α, and IL1-β expression.

We used the H&E staining to evaluated the histopathologic changes, and semiquantitative analysis of histopathologic damage in the colon was performed (Figures 3A,B). These results of CG group showed that there were no histological abnormalities. However, obvious tissue damage and inflammation were observed in the colons of the MG group. Additionally, the damage and inflammatory cell infiltration were ameliorated by AFI treatments. Then, qPCR assays were used to examine the expression of inflammatory cytokines in colon. The results showed that 2.5% DSS stimulated the expression of the cytokines IL-6, TNF-α, and IL1-β in the MG group (Figure 3D). However, these inflammatory factors were significantly reduced by AFI treatment. Additionally, the correlation heatmap showed the relationship between the bacterial family and intestinal inflammatory factors. At the phylum level, the content of the inflammatory factor IL-6 was negatively correlated with Firmicutes and positively correlated with Proteobacteria and Campilobacterota (Supplementary Figure 1). At the family level, Lactobacillaceae showed a significant negative correlation with IL-6. Bacteroidaceae, Tannerellaceae, Helicobacteraceae, Prevotellaceae and Marinifilaceae showed a significant positive correlation with IL-6. Butyricicoccaceae, Enterococcaceae and Enterobacteriaceae demonstrated a significant positive correlation with TNF-α. Corynebacteriaceae and Eggerthellaceae demonstrated a significant positive correlation with TNF and IL-1. Anaerovoracaceae showed a significant positive correlation with IL-1β (Figure 3C).

### Alpha diversity and composition of the gut microbiota

**FIGURE 4:**
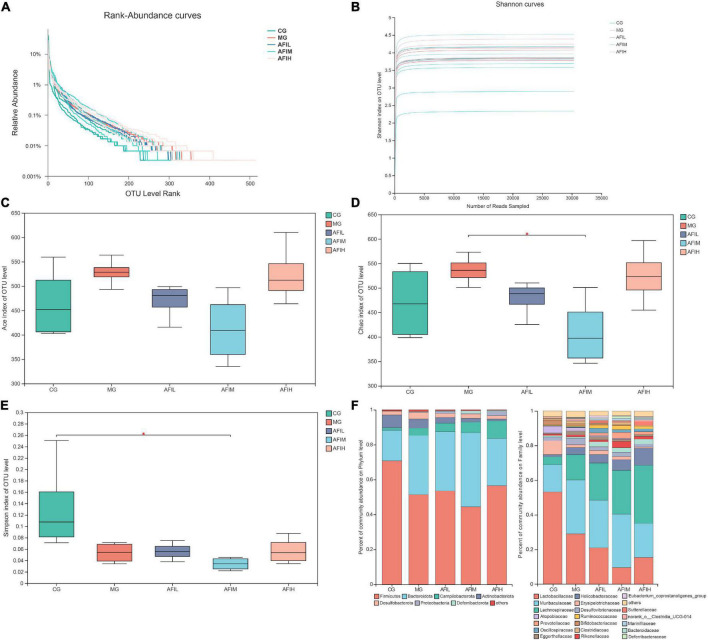
Effects of AFI on the intestinal microbiota induced by DSS. **(A)** Rank-Abundance curves of the OUT level. **(B)** Shannon dilution curves. **(C)** ACE. **(D)** Chao. **(E)** Simpson. **(F)** The composition of the gut microbiota and the community bar plot analysis at the phylum and family levels.

The rank-abundance curve was used to explain two aspects of diversity, which were species richness and community evenness. Based our result (Figure 4A), the curve of the AFIH declines gently and extends far, indicating high species diversity. However, the species diversity and richness decreased in the CG and AFIM groups. The Shannon index of the OTU level was set as the vertical axis in the rarefaction curve. The curve tends to flatten, indicating that the sample sequencing volume is sufficient, and no more OTU can be found even with the increase of data (Figure 4B). The ACE index, Chao index and Simpson index were selected for the analysis of diversity among the five groups (CG, MG, AFIL, AFIM, AFIH). Finally, the richness and diversity of the intestinal microbiota showed a tendency to increase in the MG group compared with the CG, AFIL, and AFIH groups and was significantly higher than that in the AFIM group (Figures 4C–E). Then, the top 7 dominant phyla and 20 dominant families in all samples were selected to construct a community column chart. This chart revealed that all 20 genera belong to 7 main phyla: Firmicutes, Bacteroidetes, Campilobacterota, Actinobacteriota, Desulfobacterota Proteobacteria, and Deferribacterota. Most of the dominant families in the 5 groups belonged to Firmicutes (44%-70%) and included Lactobacillaceae, Lachnospiraceae, Erysipelotrichaceae, Oscillospiraceae, Ruminococcaceae, Clostridiaceae, Clostridia_UCG-014, and Eubacterium_coprostanoligenes (Figure 4E and Supplementary Tables 2, 3). Lactobacillaceae was the predominant family in the CG group (53%), and the content of this family was much higher than that in the other groups. However, we found that AFI and DSS reduced the abundance of Lactobacillaceae. The second dominant phylum in the 5 groups was Bacteroidetes, and the dominant families included the Muribaculaceae, Bacteroidaceae, Rikenellaceae, Prevotellaceae, and Marinifilaceae families. Muribaculaceae (15.7%) was the predominant family in the CG group and belongs to Bacteroidota. These results show that the abundance of this flora is increased by AFI and DSS intervention.

### Beta diversity of the gut microbiota

**FIGURE 5:**
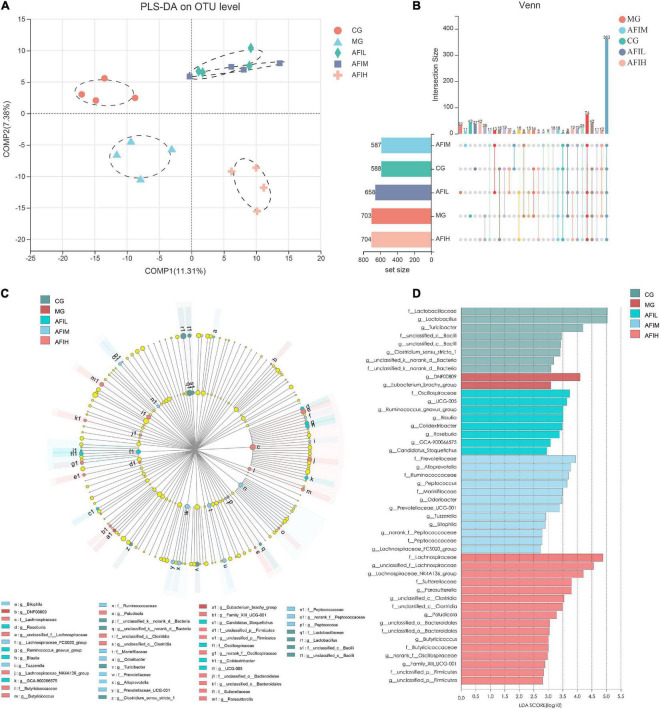
The effect of AFI on the intestinal microbiota. **(A)** Partial least squares-discriminant analysis (PLS-DA) of the OTUs in the five groups. **(B)** Advanced venn diagram (UpSet) results for the transcriptome data from the four comparisons. **(C)** Significantly different bacterial taxa at the family to genus levels. **(D)** Significant differences were tested by linear discriminant analysis effect size (LEfSe) analysis, with linear discriminant analysis (LDA) scores of >2 and p values of <0.05. The taxa enriched in the five groups showed positive LDA scores.

The beta diversity of the gut microbiota was analyzed to compare the similarity of different samples in species diversity (Figure 5). Partial least squares discriminant analysis (PLS-DA) showed that the gut microbiota of the 3 AFI groups was obviously separated from that of the other two groups, indicating that the composition of the gut microbiota was significantly different between the CG and MG groups. However, the AFIH group was obviously separated from the AFIL and AFIM groups, suggesting that AFI had a different effect on the gut microbial structure when compared with different doses (Figure 5A). The OTU distributions between different treatment groups are shown in Figure 5B. These results showed that the five groups shared the greatest number of different OTUs (363 OTUs) (Figure 5A). Additionally, four groups (AFIL, AFIM, AFIH and MG) shared 73 OTUs. Finally, LEfSe analysis identified the differentially abundant bacterial taxa in the gut microbiota between the five groups. The results showed that MG and AFI mice had lower proportions of the Lactobacillaceae family than CG mice (Supplementary Table 3). The AFI group had higher proportions of the Oscillospiraceae family than the CG and MG groups (Supplementary Table 3). We also found that the effects of different doses of AFI on the gut microbiome were different. MG and AFI mice had lower proportions of the families Lactobacillaceae than CG mice (Supplementary Table 3). We also found that the effects of different doses of AFI on the gut microbiome were different. At the family level, AFIL mice had higher proportions of Oscillospiraceae than CG and MG mice (Supplementary Table 3). However, Prevotellaceae, Ruminococcaceae, and Marinifilaceae were enriched in the AFIM group, whereas Lachnospiraceae, Lachnospiraceae, Sutterellaceae, and Butyricicoccaceae were more abundant in the AFIH group. At the genus level, Ruminococcus, Blautia, Colidextribacter, Roseburia, GCA-900066575, and Candidatus_Stoquefichus were enriched in the AFIL group. Alloprevotella, Peptococcus, Odoribacter, Prevotellaceae_UCG-001, Tuzzerella, Bilophila, and Lachnospiraceae_FCS020_group were more abundant in the AFM group, and Lachnospiraceae_NK4A136, Parasutterella, Paludicola, Butyricicoccus, and Family_XIII_UCG-001 were enriched in the AFIH group (Figures 5C,D).

## Discussion

The results of this study showed that after the mice were given DSS, the food intake and body weight were seriously reduced, the drinking water was increased, the DAI scores were reduced, and the colonic tissue damage were alleviated. Intervention with different concentrations of AFI significantly improved the physiological state of the mice, indicating that the total amount of AFI can improve the adverse reactions caused by DSS. Low-dose AFI had the best effect in reducing serum and intestinal inflammation. Decreasing NF-κB expression suppresses local inflammatory processes in the intestines. Various proinflammatory and anti-inflammatory cytokines are known to be regulated at least in part by the transcription activator NF-κB. Blocking NF-κB activation could suppress its downstream inflammatory mediators, such as IL-1β, TNF-α and IL-6, to contribute to the resolution of inflammation (21, 22). Additionally, TNF-α not only induces inflammation but also participates in regulating other immune responses in both innate and adaptive immunity, including apoptosis and cell proliferation. The present results indicated that AFI could inhibit extensive immune responses, including the inflammatory response.

Then, we examined whether the effect of AFI treatment on the mitigation of DSS-induced inflammation is related to the gut microbiota. We found that treatment with different doses of AFI partially shifted the β-diversity of the gut microbiota of DSS-treated mice, and the α-diversity (OTUs) was weakly decreased. Furthermore, both DSS and AFI treatment decreased the abundance of Firmicutes, especially the number of Lactobacillaceae. In fact, previous studies demonstrated that Lactobacillaceae, a type of probiotic, are effective inhibitors against inflammatory bowel disease development, including ulcerative colitis (23). However, the reduction in Lactobacillus abundance after AFI treatment suggests that Lactobacillus is not a key group of bacteria that play a role in AFI mitigation of inflammation. The gut microbiota results showed that the relative abundance of Oscillospira was significantly increased in the AFL group. A previous study showed that the abundance of Oscillospira is decreased in inflammatory diseases (24, 25), possibly because Oscillospira produces the short-chain fatty acid butyrate (26). Additionally, Oscillospira has been shown to produce secondary bile acids for protection against Clostridium difficile infection (27). Therefore, Oscillospira may be the key bacteria for the ability of the low-dose AFI to reduce inflammation. Additionally, the abundance of Oscillospira was also increased in the AFIM and AFIH groups compared to the CG group (Supplementary Table 3). However, Oscillospira has not been isolated and cultured (26). This study is the first to find a correlation between AFI and Oscillospira. We also noticed that the abundance of Prevotellaceae and Lachnospiraceae was significantly increased in the AFIM and AFIH groups, respectively. In fact, Prevotellaceae, a butyric acid-producing probiotic, is thought to be associated with anti-inflammatory and antioxidant activities (1, 28, 29). In addition, based on Spearman correlation analysis of intestinal inflammatory factors and intestinal microbiota, we found positive and negative correlations between the abundance of some bacteria and inflammation. The bacteria of Lachnospiraceae impact their hosts by producing short-chain fatty acids and converting primary bile acids to secondary bile acids to induce resistance against intestinal pathogens (30). The main active ingredients in AFI are neohesperidin and naringin, and previous studies have demonstrated that AFI prevents colorectal tumorigenesis by altering the gut microbiota (31, 32). In conclusion, AFI can effectively alleviate DSS-induced intestinal mucositis by inhibiting proinflammatory cytokines and regulating the overall structure and composition of the intestinal microbiota.

## Data availability statement

The data presented in the this study are deposited in the NCBI repository, https://www.ncbi.nlm.nih.gov/, accession no. PRJNA866702.

## Ethics statement

The animal study was reviewed and approved by the Animal Ethics Committee of Hunan University of Chinese Medicine.

## Author contributions

RL and TX designed this experiment and edited the manuscript. S-YC and QZ carried out animal trial and collected samples. QZ and LC detected the samples and analyzed the data. RL and XL guided the experiment and revised the manuscript. All authors have read and approved the final manuscript.

## References

[1] Cignarella F Cantoni C Ghezzi L Salter A Dorsett Y Chen L Intermittent fasting confers protection in CNS autoimmunity by altering the gut microbiota. *Cell Metab.* (2018) 27:1222. 10.1016/j.cmet.2018.05.006 29874567 PMC6460288

[2] Kaplan GG. The global burden of IBD: from 2015 to 2025. *Nat Rev Gastroenterol Hepatol.* (2015) 12:720–7. 10.1038/nrgastro.2015.150 26323879

[3] Ng SC Shi HY Hamidi N Underwood FE Tang W Benchimol EI Worldwide incidence and prevalence of inflammatory bowel disease in the 21st century: a systematic review of population-based studies. *Lancet.* (2017) 390:2769–78. 10.1016/S0140-6736(17)32448-0 29050646

[4] Loubet PS Dias TO Reis VHDT Moya AMTM Dos Santos EF Cazarin CBB. Feed your gut: functional food to improve the pathophysiology of inflammatory bowel disease. *J Funct Foods.* (2022) 93:105073.

[5] Viuda-Martos M Ruiz-Navajas Y Fernandez-Lopez J Perez-Alvarez J. Antifungal activity of lemon (*Citrus lemon* L.), mandarin (*Citrus reticulata* L.), grapefruit (*Citrus paradisi* L.) and orange (*Citrus sinensis* L.) essential oils. *Food Control.* (2008) 19:1130–8.

[6] Pultrini Ade M Galindo LA Costa M. Effects of the essential oil from *Citrus aurantium* L. in experimental anxiety models in mice. *Life Sci.* (2006) 78:1720–5. 10.1016/j.lfs.2005.08.004 16253279

[7] Yu J Wang L Walzem RL Miller EG Pike LM Patil BS. Antioxidant activity of citrus limonoids, flavonoids, and coumarins. *J Agric Food Chem.* (2005) 53:2009–14. 15769128 10.1021/jf0484632

[8] Manthey JA Guthrie N. Antiproliferative activities of citrus flavonoids against six human cancer cell lines. *J Agric Food Chem.* (2002) 50:5837–43. 10.1021/jf020121d 12358447

[9] Kim JA Park HS Kang SR Park KI Lee DH Nagappan A Suppressive effect of flavonoids from Korean *Citrus aurantium* L. on the expression of inflammatory mediators in L6 skeletal muscle cells. *Phytother Res.* (2012) 26:1904–12. 10.1002/ptr.4666 22431150

[10] Takase H Yamamoto K Hirano H Saito Y Yamashita A. Pharmacological profile of gastric mucosal protection by marmin and nobiletin from a traditional herbal medicine, Aurantii Fructus Immaturus. *Jpn J Pharmacol.* (1994) 66:139–47. 10.1254/jjp.66.139 7861659

[11] Tan WX Li Y Wang Y Zhang ZJ Wang T Zhou Q Anti-coagulative and gastrointestinal motility regulative activities of Fructus Aurantii Immaturus and its effective fractions. *Biomed Pharmacother.* (2017) 90:244–52. 10.1016/j.biopha.2017.03.060 28363170

[12] Nakajima A Yamakuni T Haraguchi M Omae N Song SY Kato C Nobiletin, a citrus flavonoid that improves memory impairment, rescues bulbectomy-induced cholinergic neurodegeneration in mice. *J Pharmacol Sci.* (2007) 105:122–6. 10.1254/jphs.sc0070155 17895593

[13] Cao R Wu X Guo H Pan X Huang R Wang G Naringin exhibited therapeutic effects against DSS-induced mice ulcerative colitis in intestinal barrier-dependent manner. *Molecules.* (2021) 26:6604. 10.3390/molecules26216604 34771012 PMC8588024

[14] Hu J Huang H Che Y Ding C Zhang L Wang Y Qingchang Huashi formula attenuates DSS-induced colitis in mice by restoring gut microbiota-metabolism homeostasis and goblet cell function. *J Ethnopharmacol.* (2021) 266:113394. 10.1016/j.jep.2020.113394 32941971

[15] Cooper HS Murthy SN Shah RS Sedergran DJ. Clinicopathologic study of dextran sulfate sodium experimental murine colitis. *Lab Invest.* (1993) 69:238–49. 8350599

[16] Chen S Zhou Y Chen Y Gu J. fastp: an ultra-fast all-in-one FASTQ preprocessor. *Bioinformatics.* (2018) 34:i884–90. 10.1093/bioinformatics/bty560 30423086 PMC6129281

[17] Magoc T Salzberg SL. FLASH: fast length adjustment of short reads to improve genome assemblies. *Bioinformatics.* (2011) 27:2957–63. 10.1093/bioinformatics/btr507 21903629 PMC3198573

[18] Stackebrandt E Goebel BM. Taxonomic note: a place for DNA-DNA reassociation and 16S rRNA sequence analysis in the present species definition in bacteriology. *Int J Syst Evol Microbiol.* (1994) 44:846–9.

[19] Edgar RC. UPARSE: highly accurate OTU sequences from microbial amplicon reads. *Nat Methods.* (2013) 10:996–8. 10.1038/nmeth.2604 23955772

[20] Wang Q Garrity GM Tiedje JM Cole JR. Naive Bayesian classifier for rapid assignment of rRNA sequences into the new bacterial taxonomy. *Appl Environ Microbiol.* (2007) 73:5261–7. 10.1128/AEM.00062-07 17586664 PMC1950982

[21] Lawrence T Bebien M Liu GY Nizet V Karin M. IKKalpha limits macrophage NF-kappaB activation and contributes to the resolution of inflammation. *Nature.* (2005) 434:1138–43. 10.1038/nature03491 15858576

[22] Taniguchi K Karin M. NF-kappaB, inflammation, immunity and cancer: coming of age. *Nat Rev Immunol.* (2018) 18:309–24. 10.1038/nri.2017.142 29379212

[23] Dordevic D Jancikova S Vitezova M Kushkevych I. Hydrogen sulfide toxicity in the gut environment: meta-analysis of sulfate-reducing and lactic acid bacteria in inflammatory processes. *J Adv Res.* (2021) 27:55–69. 10.1016/j.jare.2020.03.003 33318866 PMC7728594

[24] Zhu L Baker SS Gill C Liu W Alkhouri R Baker RD Characterization of gut microbiomes in nonalcoholic steatohepatitis (NASH) patients: a connection between endogenous alcohol and NASH. *Hepatology.* (2013) 57:601–9. 10.1002/hep.26093 23055155

[25] Walters WA Xu Z Knight R. Meta-analyses of human gut microbes associated with obesity and IBD. *FEBS Lett.* (2014) 588:4223–33. 25307765 10.1016/j.febslet.2014.09.039 PMC5050012

[26] Konikoff T Gophna U. Oscillospira: a central, enigmatic component of the human gut microbiota. *Trends Microbiol.* (2016) 24:523–4. 10.1016/j.tim.2016.02.015 26996766

[27] Keren N Konikoff FM Paitan Y Gabay G Reshef L Naftali T Interactions between the intestinal microbiota and bile acids in gallstones patients. *Environ Microbiol Rep.* (2015) 7:874–80. 10.1111/1758-2229.12319 26149537

[28] Huang P Jiang A Wang X Zhou Y Tang W Ren C NMN maintains intestinal homeostasis by regulating the gut microbiota. *Front Nutr.* (2021) 8:714604. 10.3389/fnut.2021.714604 34395502 PMC8358781

[29] Teng T Clarke G Maes M Jiang Y Wang J Li X Biogeography of the large intestinal mucosal and luminal microbiome in cynomolgus macaques with depressive-like behavior. *Mol Psychiatry.* (2022) 27:1059–67. 10.1038/s41380-021-01366-w 34719692 PMC9054659

[30] Sorbara MT Littmann ER Fontana E Moody TU Kohout CE Gjonbalaj M Functional and genomic variation between human-derived isolates of Lachnospiraceae reveals inter- and intra-species diversity. *Cell Host Microbe.* (2020) 28:134. 10.1016/j.chom.2020.05.005 32492369 PMC7351604

[31] Powell F Rothwell L Clarkson M Kaiser P. Development of reagents to study the Turkey’s immune response: cloning and characterisation of two Turkey cytokines, interleukin (IL)-10 and IL-13. *Vet Immunol Immunopathol.* (2012) 147:97–103. 10.1016/j.vetimm.2012.03.013 22521280 PMC7127247

[32] Gong Y Dong R Gao X Li J Jiang L Zheng J Neohesperidin prevents colorectal tumorigenesis by altering the gut microbiota. *Pharmacol Res.* (2019) 148:104460. 10.1016/j.phrs.2019.104460 31560944

