# Supplementary material for: The Aurantii Fructus Immaturus flavonoid extract alleviates inflammation and modulate gut microbiota in DSS-induced colitis mice
Source: Front Nutr. 2022 Oct 5;9:1013899. doi: 10.3389/fnut.2022.1013899 (PMC9581122; doi:10.3389/fnut.2022.1013899)
Supplement: Supplementary file 1 [file Data_Sheet_1.pdf]

Supplementary Material

1 Supplementary Figures and Tables

1.1 Supplementary Figures

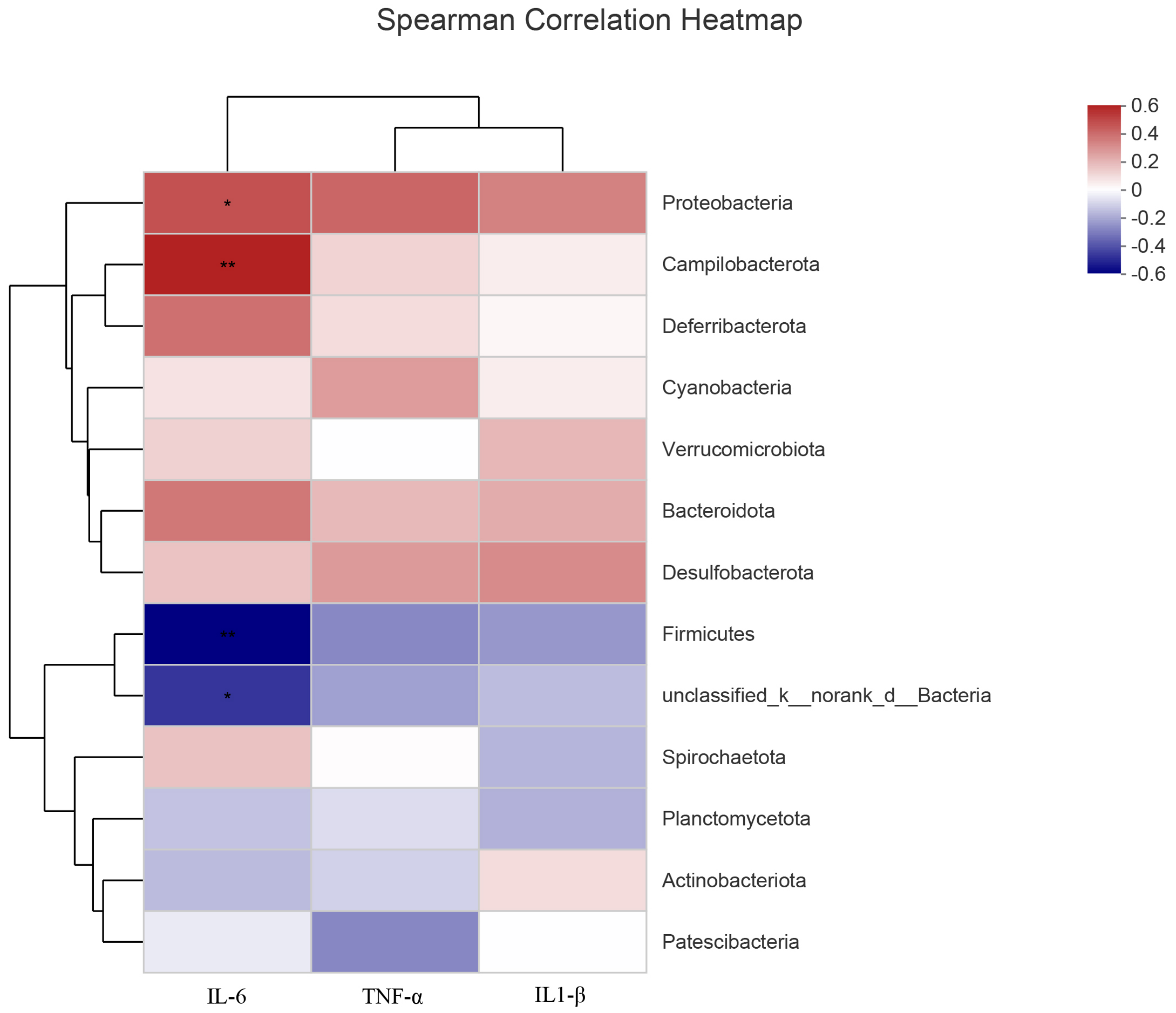

Supplementary Figure 1. Supplementary Figure 1. The correlation heatmap of IL-6, TNF-a, IL-1 and microbiota on phylum level.

1.2 Supplementary Tables

Supplementary table 1. Primers used in the paper.

Primers used for CDS amplification	
name	Sequences
IL-1β-F	CACTACAGGCTCCGAGATGAACAAC
IL-1β-R	TGTCGTTGCTTGGTTCTCCTTGTAC
IL-6-F	CTCCCAACAGACCTGTCTATAC
IL-6-R	CCATTGCACAACTCTTTTCTCA
TNF-α-F	ATGTCTCAGCCTCTTCTCATTC
TNF-α-R	GCTTGTCACTCGAATTTTGAGA

Supplementary Table 2. The differences among 5 grops gut microbiota of mice at the phylum levels

Phylum	CG	MG	AFIL	AFIM	AFIH
Firmicutes	0.707305816	0.513747857	0.53513616	0.443978307	0.56444514
Bacteroidota	0.175812673	0.338627522	0.338709943	0.423925227	0.271717988
Campilobacterota	0.013451141	0.041672161	0.049139523	0.061857115	0.099655479
Actinobacteriota	0.073684558	0.052914414	0.033949294	0.021841619	0.010813662
Desulfobacterota	0.021907556	0.036941184	0.020959713	0.02405875	0.018585982
Proteobacteria	0.001038507	0.002233615	0.011555453	0.004887577	0.024767572
Deferribacterota	0.000684096	0.003395754	0.004920546	0.013632467	0.00684096
others	0.006115653	0.010467493	0.005629368	0.005818937	0.003173216

Supplementary Table 3. The differences among 5 grops gut microbiota of mice at the genus levels

Phylum	Familly	CG	MG	AFIL	AFIM	AFIH
Firmicutes	Lactobacillaceae	0.532136028	0.290847949	0.210396611	0.0967048	0.154630423
Bacteroidota	Muribaculaceae	0.157498681	0.311173019	0.275352763	0.308073981	0.197497692

Firmicutes	Lachnospiraceae	0.044688778	0.146544903	0.214517671	0.253214427	0.334696031
Campilobacterota	Helicobacteraceae	0.013451141	0.041672161	0.049139523	0.061857115	0.099655479
Firmicutes	Erysipelotrichaceae	0.080270012	0.015989714	0.023745549	0.017226032	0.005456284
Desulfobacterota	Desulfovibrionaceae	0.021907556	0.036941184	0.020959713	0.02405875	0.018585982
Bacteroidota	Bacteroidaceae	0.007994857	0.008209152	0.028847422	0.027462746	0.02779243
Bacteroidota	Rikenellaceae	0.008373994	0.011143347	0.010459251	0.036751615	0.015866082
Actinobacteriota	Eggerthellaceae	0.005753	0.022995516	0.019781089	0.011670843	0.010434525
Actinobacteriota	Atopobiaceae	0.039595147	0.018297508	0.006494791	0.005654095	0.000280232
Bacteroidota	Prevotellaceae	0.000733549	0.001862719	0.0140693	0.034814717	0.016319399
Firmicutes	Oscillospiraceae	0.002225373	0.005241989	0.027965515	0.015486944	0.016739747
Firmicutes	Ruminococcaceae	0.004491956	0.006090927	0.017250758	0.023168601	0.015033628
Actinobacteriota	Bifidobacteriaceae	0.02833641	0.011481274	0.007549782	0.004516682	9.07E-05
Firmicutes	Clostridiaceae	0.008637742	0.0143825	0.005027694	0.013896215	0.003684228

Proteobacteria	Sutterellaceae	0.000989054	0.001038507	0.011126863	0.004615587	0.023926876
Firmicutes	Clostridia_UCG-014	0.008621258	0.012420876	0.005489252	0.006577212	0.008299815
Bacteroidota	Marinifilaceae	0.000197811	0.003338059	0.007071739	0.012132401	0.007533298
Deferribacterota	Deferribacteraceae	0.000684096	0.003395754	0.004920546	0.013632467	0.00684096
Firmicutes	Eubacterium_coprostanoligenes_group	0.000263748	0.001681393	0.013475867	0.001467097	0.005555189
	others	0.033149809	0.03525155	0.026358301	0.027017671	0.031081037
